# Overview of the *Ruspolia* Plant Genus: Insights into Its Chemical Diversity and Biological Potential

**DOI:** 10.3390/life15020221

**Published:** 2025-02-02

**Authors:** Christian Bailly, Gérard Vergoten

**Affiliations:** 1 Faculté de Pharmacie, Institut de Chimie Pharmaceutique Albert Lespagnol (ICPAL), University of Lille, 3 rue du Professeur Laguesse, BP-83, 59006 Lille, France; 2 UMR9020-U1277—CANTHER—Cancer Heterogeneity Plasticity and Resistance to Therapies, CHU Lille, Inserm, CNRS, University of Lille, 59000 Lille, France; 3 OncoWitan, Scientific Consulting Office, 59290 Wasquehal, France; 4 Inserm, U1286–INFINITE—Lille Inflammation Research International Center, ICPAL, University of Lille, 3 rue du Professeur Laguesse, 59000 Lille, France

**Keywords:** hypercratine, justicialoside, pyrrolidine alkaloids, *Ruspolia* plants, ruspolinone

## Abstract

The genus *Ruspolia* refers to a small group of plants in the Acanthaceae family, with two dominant species *R. decurrens* and *R. hypocrateriformis* essentially distributed in tropical parts of Africa. Decoctions from these plants are used in folk medicine for the treatment of a few human pathologies but the active ingredients at the origin of the bioactivities have been little studied. Here, we give an insight into the main phytochemicals of the *Ruspolia* species published in the literature so far and their pharmacological properties. The flavone glycosides justicialosides A-B likely serve as antioxidant agents and free radical scavengers. Several pyrrolidine alkaloids have been isolated from these *Ruspolia* species, notably (nor)ruspolinone and a few related products. These molecules have attracted the interest of medicinal chemists, with different synthetic routes leading to ruspolinone and analogues. There are versatile operating procedures to synthesize (nor)ruspolinone isomers. Despite these chemical efforts, the pharmacology of ruspolinone remains largely unknown. A few other *Ruspolia* alkaloids have been isolated, notably the rare bispyrrolidine benzodioxin alkaloid hypercratine, possibly acting as a ligand of β2-adrenergic receptors. A phytochemical survey of the *Ruspolia* species sheds light on the diversity of products in this family to promote further investigations into the mechanism of action of ruspolinone and related natural products.

## 1. Introduction

**Figure 1:**
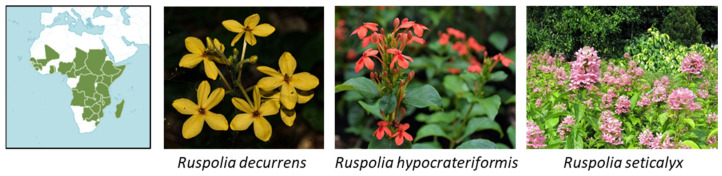
Distribution of *Ruspolia* species in Africa (maps defined from World Flora Online (www.worldfloraonline.org, accessed on 3 January 2025) and selected views of the flowers of three *Ruspolia* species.

Plants from the genus *Ruspolia* in the Acanthaceae family are distributed essentially in tropical regions of Africa, from Mali to Sudan in the north, and from Angola to the Republic of Mozambique in the south, as well as the island of Madagascar (Figure 1). The name *Ruspolia* was given by the German mycologist and botanist Gustav Lindau (1866–1923). It pays homage to the Italian botanist Francesco Maria Marescotti Ruspoli (1672–1731). Parenthetically, the name Ruspolia should not be confused with that of the three plant species *Lopriorea ruspolii* (family: Amaranthaceae), *Moringa ruspoliana* Engl. (family: Moringaceae), and *Pterodiscus ruspolii* Engl. (family: Pedaliaceae), named after another Italian naturalist, Prince Eugenio Ruspoli (1866–1893).

The *Ruspolia* genus includes five little-known species with an accepted name (Table 1). The major species is *R. decurrens*, a subshrub mainly found in central and south-tropical Africa. It grows primarily in the seasonally dry tropical biome. This vascular plant producing nice yellow flowers (Figure 1) can be found in riverine forests, shady places in woodland and rocky hills and on termite mounds, notably in Zambia and Zimbabwe [1,2]. The species *R. hypocrateriformis* (Vahl), also known as *Justicia hypocrateriformis* Vahl, is a medicinal plant used to prepare a herbal remedy for diarrhea in Cameroon folk medicine [1,2]. In the rural area of Bui Division of Cameroon, a decoction from the leaves and stems of *R. hypocrateriformis* (locally named “kifu ke menseh”) is used in combination with two other plants to treat anemia, hemiplegia, and neuralgia [3,4]. This fast-growing evergreen plant shows nice pink to red flowers producing a large amount of nectar largely consumed by butterflies. *Ruspolia hypocrateriformis* (Vahl) is not to be confused with the totally distinct species *Rivea hypocrateriformis* (Desr.) Choisy, an edible plant found in India [5]. The species *R. australis* is native to Tanzania and South Africa. It is a tall shrub known as red mock-plumbago, also producing scarlet red flowers. The species *Ruspolia seticalyx* is native to east and south tropical Africa, whereas *Ruspolia paniculata* is found essentially in Madagascar. There is also a species called *Ruspolia humbertii* Benoist (family: Acanthaceae), also originating in Madagascar, but it has an unchecked status in the World Flora Online repertoire. The main species in the genus is *R. hypocrateriformis,* which also presents a nutritional potential due to its content in mineral elements, macro-nutrients (carbohydrates and fibers), and vitamins, such as vitamin C (1.22 g/100 g) [6].

It is worth noting that the name *Ruspolia* refers also to a genus of insects (family: Tettigoniidae; order: Orthoptera) including the two species *R. differens* and *R. nitidula*. The former is an edible grasshopper, very popular as a food supplement in central and eastern Africa [7]. The fortification of sorghum and wheat with longhorn *R. differens* powder (RDP) is considered for use as a supplementary food used to prevent protein-energy malnutrition in children. RDP-fortified biscuits are being commercialized [8]. The latter is also an edible grasshopper, used as a protein source in Uganda [9,10]. The grasshopper *Ruspolia nitidula* has expanded its area of distribution in western and central Europe in recent decades [11]. The *Ruspolia* species of insects can also be seen in India (Kashmir) [12]. Bioactive compounds can be found in edible insects, including for example antioxidant compounds isolated from *R. differens* [13]. There are a few others insect species (e.g., *R. dubia*, *R. Yunnana*) [14,15] but here the analysis will focus on the *Ruspolia* plant species only.

Among *Ruspolia* plant species, *R. hypocrateriformis* is the most important species from a medicinal viewpoint. In Cameroon, a decoction of the leaves of this plant is used for the treatment of anemia and fever associated with malaria, and for the management of diarrhea [16]. As mentioned above, *R. hypocrateriformis* is used also in combination with other plants to treat hemiplegia and neuralgia [3,4]. Ethnobotanical practices have been rarely reported with the other *Ruspolia* species. Nevertheless, several bioactive natural products have been discovered from these plants. The present analysis discusses the pharmacological properties of *Ruspolia* plant extracts to highlight their potential as a valuable source of natural remedies. The primary objective of the review is to provide an overview of the various phytochemicals isolated from *Ruspolia* plants, the associated chemistry, and their bioactive properties. The major objective of the research is to fill knowledge gaps in *Ruspolia* phytochemistry and pharmacology, to encourage further studies of these families of neglected medicinal plants.

## 2. Flavone Glycosides from *Ruspolia*

**Figure 2:**
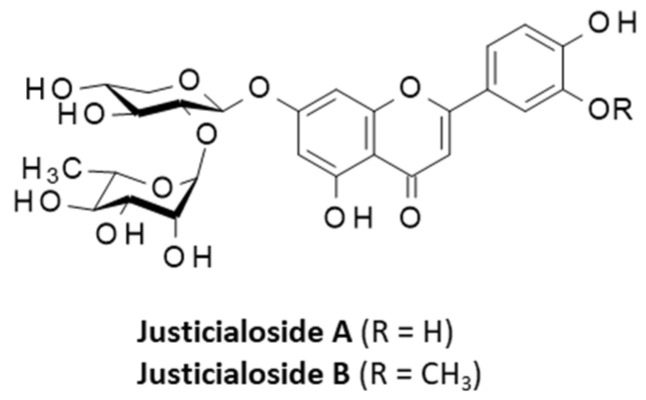
Structures of two flavone glycosides isolated from *Ruspolia hypocrateriformis*.

An ethanol leaf extract of *R. hypocrateriformis* has been shown to contain common phenolic compounds such as gallic acid and ferulic acid, and a few flavonoids such as quercetin. These chemicals contribute to the antioxidant properties of the plant extract [17]. This plant also contains flavonoid *O*-glycosides, such as the two glycosylated luteolin derivatives: luteolin 7-*O*-β-D-apiofuranosyl-(1→2)-β-D-xylopyranoside and luteolin 7-*O*-[-βD-glucopyranosyl-(1→2)-α-L-rhamnosyl-(1→6)]-βD-glucopyranoside. In addition, two specific glycosides have been isolated from the plant leaves and named justicialosides A and B. They bear a luteolin or a chrysoeriol flavonoid core linked to a disaccharide unit (7-*O*-*α*-L-rhamnopyranosyl-(1→2)-*β*-D-xylopyranoside) (Figure 2). Their names derive from *Justicia hypocrateriformis*, the synonym for *R. hypocrateriformis* [18].

These different flavonoids contribute to the antioxidant activity of the plant extract, and possibly to anti-anemia and antidiarrheal properties as well [2,6,19]. The glycoside moiety of the flavonoid contributes to its antioxidant action. Chrysoeriol is a multipotent flavone with anticancer, anti-inflammatory, antibacterial, antifungal, anti-osteoporosis, insecticide, and neuroprotective activities [20]. Chrysoeriol glycoside has been shown to inhibit the production superoxide anion by the xanthine/xanthine oxidase system more effectively than the aglycone. Similarly, the glycoside is a more efficient scavenger of 1,1-diphenyl-2-trinitrophenylhydrazine (DPPH) free radicals than the aglycone (chrysoeriol) itself [21]. A methanolic fraction of *R. hypocrateriformis* has revealed a modest antimalarial activity associated with inhibition of β-hematin formation (IC_50_ = 170 μg/mL) [22]. Justicialosides are phytochemicals specific to *R. hypocrateriformis*. Nevertheless, justicialoside A has been identified also from the leaves and stem bark of the African medicinal plant *Pseudospondias microcarpa* (A. Rich.) Engl. (Anacardiaceae) [23]. The pharmacological properties of the two justicialosides have not been specifically investigated. It would be useful to study their stress-reducing properties because a very similar flavone (acacetin) bearing exactly the same diglycoside moiety has been shown to reduce stress response and to promote longevity in a *Caenorhabditis elegans* model system. Notably, the product enhanced the levels of the antioxidant enzymes superoxide dismutase and catalase [24].

## 3. Pyrrolidine Alkaloids from *Ruspolia*

### 3.1. Isolation of the Natural Products

**Figure 3:**
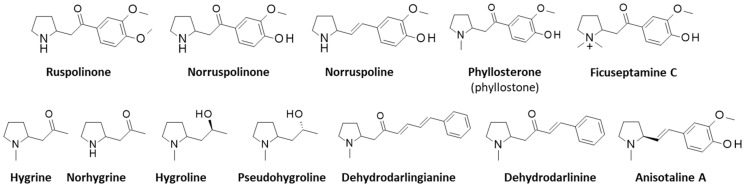
Structures of pyrrolidine alkaloids related to ruspolinone.

Ruspolinone, norruspolinone, and norruspoline are three pyrrolidine alkaloids isolated from *R. hypocrateriformis* [25]. There are also derivatives, such as N-methylruspolinone [26,27]. The pyrrolidinyl moiety of (nor)ruspolinone is reminiscent of that of the coca leaf alkaloids (nor)hygrine and the related alkaloids hygroline and pseudohygroline from the plants *Carallia brachiata*, *Erythroxylum coca*, *Schizanthus hookeri*, and *Schizanthus tricolor* [28,29,30,31]. They belong to a group of naturally occurring 2-(acylmethylene)pyrrolidine alkaloids which also includes dehydrodarlinine and dehydrodarlingianine derived from the plant *Darlingia darlingiana* found in Queensland (Australia) [26,32,33]. Phyllosterone (also named phyllostone) is a pyrrolidinyl-acetophenone derivative from *Crytopcarya phyllostemon* and is very similar to norruspoline [34]. This is also the case for the alkaloid ficuseptamine C isolated from the leaves of *Ficus septica* [35] (Figure 3). Norruspoline is structurally close to the pyrrolidine alkaloid anisotaline A (N-methyl norruspoline) isolated from the roots of the Chinese plant *Anisodus tanguticus* (Maxim.) Pascher. This compound has shown a very modest effect on the viability of human umbilical vein endothelial cells (HUVECs) in vitro [36]. These different pyrrolidine alkaloids have been described but they have been neglected from a pharmacological standpoint.

Ruspolinone was initially isolated from the *Ruspolia* species but later the alkaloid was found in a few other plants, notably the fruits of the Vietnamese plant *Boehmeria holosericea* Blume (Urticaceae) [37], the leaves of *Tephrosia pentaphylla* (Roxb.) G.Don. (Fabaceae) [38], and the aerial parts of the invasive vine *Vincetoxicum rossicum* Kelopow [39]. This phenacylpyrrolidine has also been identified from endophytic fungi: (i) from a culture of *Fusarium equiseti*, initially isolated from the leaves of *Ocimum gratissimum* L. (clove basil, Lamiaceae) [40] and (ii) an endophytic fungus isolated from leaves of *Newbouldia laevis* [41]. Ruspolinone is generally considered as an intermediate in the biosynthesis of more complex alkaloids.

### 3.2. Total Synthesis of Ruspolinone and Analogues

**Scheme 1:**
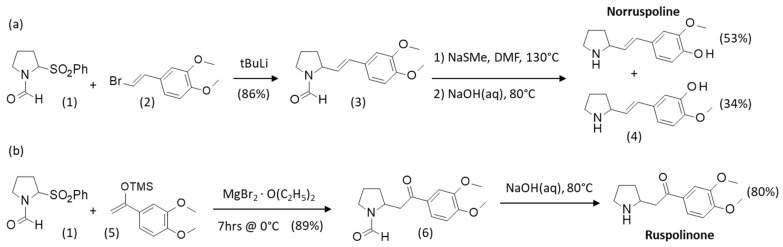
Synthetic routes to (**a**) norruspoline and (**b**) ruspolinone. Adapted from [43,44].

Different methods have been proposed for the synthesis of ruspolinone and related pyrrolidine alkaloids. The chemistry of this group of pyrrolidine alkaloids has been well studied, in particular for (nor)hygrine-type natural products [42,43]. The synthesis of ruspolinone analogues has been less developed but, nevertheless, different access routes have been described. A total synthesis of both ruspolinone and norruspoline from 2-phenysulfonyl-piperidine derivatives has been proposed (Scheme 1). Norruspoline was obtained from the sulphonyl derivative (1) in the presence of β-bromo-3,4-dimethoxystyrene (2) and butyl lithium. The reaction afforded the ethenyl-pyrrolidine derivative (3) with a yield of 86% and the subsequent two-step deprotection of this intermediate with Na thiomethoxide in dry DMF followed with a treatment with aqueous sodium hydroxide (NaOH) afforded norruspoline as a major product with a yield of 53% and its isomer (4) (Scheme 1a). The synthesis of ruspolinone proceeded similarly from the intermediate (6), itself obtained from (1) in the presence of 1,2-dimethoxy-4-[1-[(1,2-dimethylethyl)silyloxy]ethenylbenzene (5) and magnesium bromide etherate at 0 °C. In this case, the final treatment of (6) with aqueous NaOH at reflux afforded ruspolinone with a yield of 80% (Scheme 1b) [44,45].

**Scheme 2:**
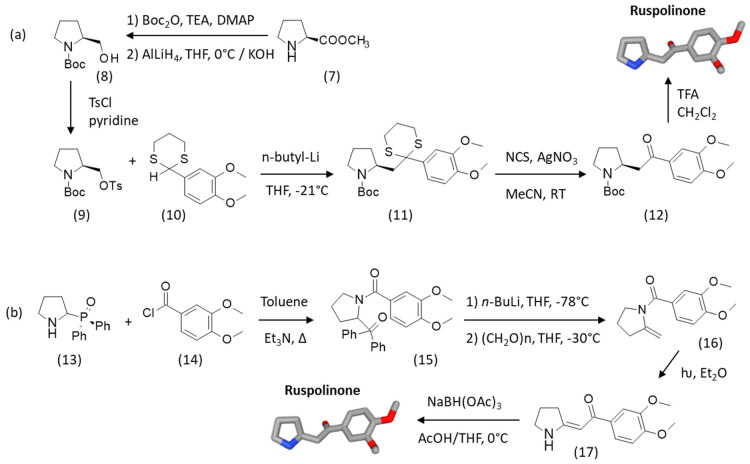
Alternative synthetic routes to ruspolinone. (**a**) Starting from L-proline methyl ester or (**b**) from 2-diphenylphosphinoyl pyrrolidine. Adapted from [45,47].

An alternative procedure was reported for the synthesis of ruspolinone starting from L-proline methyl ester (7), as depicted in Scheme 2. This seven-step procedure afforded the product with an overall yield of 26%, together with the related natural product phyllosterone (phyllostone) [46]. Compound (7) is converted into N-Boc prolinol (8) and then the O-tosylated derivative (9). Coupling of (9) with 2-(3′,4′-dimethoxyphenyl)-1,3-dithiane (10) in cold tetrahydrofuran (THF, −21 °C) in the presence of n-butyllithium in hexane afforded compound (11), purified by flash chromatography. The reaction of the dithiane (11) with N-chlorosuccinimoide (NCS) and silver nitrate in aqueous acetonitrile afforded the carbamate N-Boc-ruspolinone (12). Finally, the removal of the Boc protecting with trifluoroacetic acid (TFA) gave ruspolinone (Scheme 2a).

An alternative process to obtain ruspolinone consists in synthesizing an enaminoketone intermediate which is then selectively reduced in the presence of sodium triacetoxyborohydride (NaBH(OAc)_3_, also known as sodium triacetoxyhydroborate or STAB). The pyrrolidine enaminoketone moiety combines the nucleophilicity of the enamine with the eletrophilicity of the enone moiety [47]. 2-Diphenylphosphinoyl pyrrolidine (13) was treated with the substituted acyl chloride derivative (14) to afford the phosphorylated N-acylamine (15) with a yield of 75%. Treatment of phosphorylated amide (15) with n-butyllithium (n-BuLi) in THF and then with a pre-cooled THF solution of freshly depolymerized paraformaldehyde led to the desired pyrrolidine derivative (16) with a moderate yield of 55%. The irradiation at 254 nm of a deaerated solution of (16) in ether for about 30 min led to vinylogous amide (17). The final chemoselective reduction of the C=C unit of this enaminone (17) with sodium triacetoxyborohydride in a 3:1 mixture of acetic acid (AcOH) and THF at 0 °C led efficiently (91% yield) to the desired product ruspolinone. The same procedure can be adapted to the synthesis of both the corresponding piperidine and pyrrolidine derivatives [48].

**Scheme 3:**
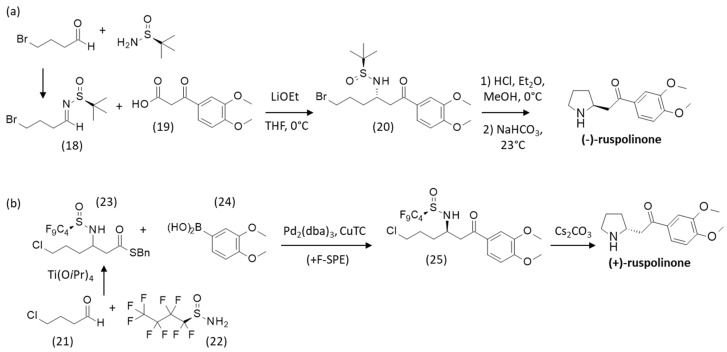
Synthetic routes to (**a**) (−)- and (**b**) (+)-ruspolinone. Adapted from [49].

More recently, Sirvent and coworkers have accomplished the synthesis of (-)-ruspolinone from *N*-*tert*-butanesulfinyl bromoaldimine (18) in the presence of a β-keto acid (19), affording the β-amino ketone derivative (20) in 82% yield. The imine (18) derived from 4-bromobutanal and (*S*)-*tert*-butanesulfinamide in toluene. After removal of the sulfinyl group of (20) under acidic conditions, the compound was transformed into (-)-ruspolinone in 92% yield, as represented in Scheme 3a [49]. A totally distinct synthetic route to (+)-ruspolinone from 4-chlorobutanal (21) and nonafluorobutane-1-sulfinamide (22) in the presence of titanium(IV) isopropoxide [Ti(O*i*Pr)_4_] as a dehydrating agent, has been proposed, as depicted in Scheme 3b. The procedure involves the synthesis of a fluorous-tagged intermediate, separated via a process known as fluorous solid-phase extraction (F-SPE) which avoids column chromatography over multiple steps. The initial step leading to the sulfinamide derivative (23) is very efficient (98% yield). A Liebeskind−Srogl cross-coupling reaction of the thioester (23) with the boronic acid derivative (24) yielded the β-amino ketone (25) after F-SPE. Finally, the *N*-sulfinyl deprotection in the presence of cesium carbonate and intramolecular rearrangement afforded (+)-ruspolinone (Scheme 3b). A similar activated *N*-perfluorobutanesulfinamide intermediate was used to synthesize (+)-ruspolinone and the antibacterial alkaloid (+)-negamycin and the antidiabetic drug (-)-sitagliptin [50].

Altogether, these different chemical processes underlined the attractiveness of the ruspolinone in terms of synthetic chemistry. There are multiple routes to access the product and derivatives. The process initially proposed by Brown and coworkers remains a straightforward and efficient route [44,45]. The (nor)ruspolinone chemistry has been well developed. Unfortunately, the same cannot be said for the pharmacology of these compounds, largely neglected until now.

### 3.3. Other Ruspolia Alkaloids

**Figure 4:**
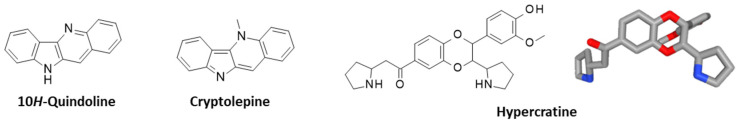
Chemical structures of 10*H*-quindoline, cryptolepine, and hypercratine (with a molecular model).

10*H*-quindoline (10*H*-indolo [3,2-b]quinoline), a tetracyclic alkaloid found in the *Justicia* species, particularly in *J. betonica* [51,52], has also been identified and isolated from the extract of the leaves of *R. hypocrateriformis* [17]. The pharmacological properties of 10*H*-quindoline have not been specifically investigated but the compound bears a close structural analogy with the well-known alkaloid cryptolepine (Figure 4) which exhibits many types of activities, including anti-bacterial, anti-fungal, anti-hyperglycemic, antidiabetic, anti-inflammatory, anti-hypotensive, antipyretic, and antimuscarinic properties [53]. Cryptolepine is a cytotoxic DNA-intercalating inhibitor of topoisomerase II [54,55,56]. It is therefore conceivable that 10*H*-quindoline can function also as a topo II poison, although other cytotoxic 10*H*-quindoline derivatives have revealed a mechanism of action independent from topoisomerase II poisoning [57]. Quindoline and derivatives have been shown to stabilize quadruplex DNA and to inhibit telomerase [58,59].

**Figure 5:**
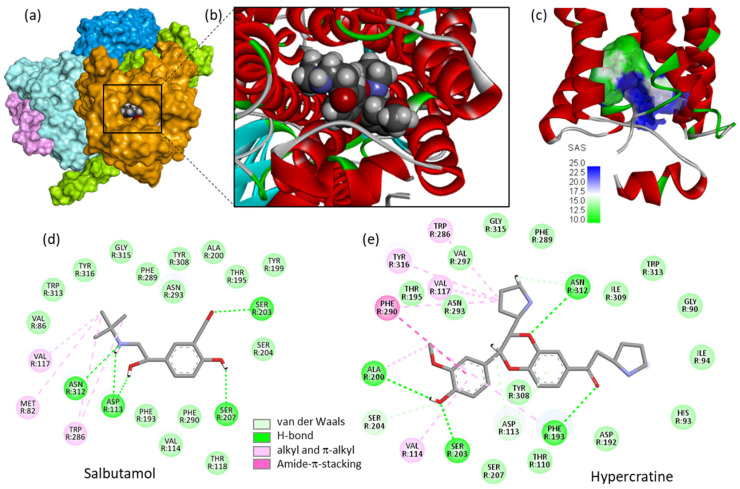
Molecular model of hypercratine (compound CID: 177183) bound to the β2-adrenergic receptor (β2AR). (**a**) A surface of hypercratine bound to β2AR (PDB structure: 7DHR). (**b**) Close-up view of the ligand−protein interface. (**c**) The solvent-accessible surface (SAS) surrounding the drug binding zone (color code indicated). (**d**,**e**) Binding map contacts for salbutamol and hypercratine bound to β2AR (color code indicated). The docking analysis was performed as previously described with other protein−drug complexes [68,69].

A rare bispyrrolidine alkaloid named hypercratine (Figure 4) was isolated from the roots of *R. hypocrateriformis* [60]. It is a benzodioxin derivative equipped with two pyrrolidinyl moieties, also identified in the leaves of the medicinal plant *Justicia flava* Vahl (Acanthaceae), possibly contributing to the tocolytic activity of the plant extract [61]. *Justicia flava* leaf extracts have been shown to potently inhibit uterine contractility in both a pregnant and a non-pregnant mouse uterus [62]. Hypercratine may contribute to the regulation of myometrial contractility. Its precise mechanism of action remains unknown at present but a binding to β2-adrenergic receptors (β2AR) is conceivable, by analogy with structurally related tocolytic agents such as salbutamol, isoproterenol, and terbutaline acting as β2AR agonists [63,64]. The targeting of the G protein-coupled receptor (GPCR) β2AR with the partial agonist salbutamol contributes to the successful use of this drug in treating asthma and chronic obstructive pulmonary disease (COPD). Hypercratine appears to be well adapted to bind tightly to β2AR, as illustrated in the molecular model shown in Figure 5. A docking analysis performed with β2AR bound to salbutamol (PDB: 7DHR) [65] revealed that the natural product can fit very well into the large salbutamol-binding cavity, engaging H-bonds or van der Waals contacts with much the same amino acid residues. A large set of molecular contacts stabilizes the hypercratine-β2AR complex and the two pyrrolidine units contribute significantly to its stability. The docking analysis suggests that the hypercratine-β2AR complex is more stable than that formed with the known β2AR ligands. The calculated empirical energy of interaction (ΔE) is largely more negative with hypercratine compared with terbutaline, ritodrine, isoproterenol, or salbutamol (Table 2). Hypercratine seems to be a bona fide β2AR ligand but its pharmacological effects, as an agonist or antagonist, remain to be characterized. Its benzodioxin structure is also reminiscent to that of the α2-adrenoceptor antagonists idazoxan and the antipsychotic derivative RX 821002 (2-methoxyidazoxan), which possesses high and selective affinities for D2-like and 5-HT(1A) receptors [66,67]. Hypercratine is a rare alkaloid totally neglected at present. This atypical natural product warrants further investigation.

## 4. Discussion

Acanthaceae is a large plant family with over 2500 species, found primarily in subtropical and tropical regions. Several species of this family have been used traditionally to treat a variety of diseases and inflammatory conditions, including gastrointestinal and cardiovascular ailments. The family includes important and well-known genera, such as the genus *Justicia* with more than 600 species [1], and the genera *Barleria* [70], *Blepharis* [71], and *Acanthus* [72]. The family also comprises little-known genera such as Ruspolia Lindau., which belongs to *Justicieae* Dumort., the most taxonomically complex tribe in Acanthaceae [73]. Within the tribe *Justicieae*, the subtribe *Graptophyllinae* T. Anderson comprises 27 genera, including Ruspolia Lindau [74].

Plants from the genus Ruspolia have been little investigated thus far. The two main species R. decurrens and R. *hypocrateriformis* are emblematic of this family of plants essentially found in Africa. Different alkaloids and flavones have been isolated from these species, notably the leading product ruspolinone (Table 3). This small pyrrolidine alkaloid and its diverse analogues have attracted interest from medicinal chemists essentially, but the associated pharmacology remains largely unknown at present. There are efficient routes to access ruspoline and (nor)ruspolinone, but little mechanistic and pharmacological information is available. Ruspolinone is readily accessible and represents an intermediate in the synthesis of more complex indolizidine alkaloids, notably in the antofine series [75]. Its chemistry has been largely investigated, but the biological properties of this alkaloid remain to be discovered. It is high time to investigate the pharmacological effects of this series of pyrrolidine alkaloids.

Ruspolinone seems to be an intermediate in the biosynthesis of hypercratine, a unique alkaloid isolated from *Ruspolia*. This rare benzodioxin alkaloid has been described in a single study, together with a few acetylated and methoxylated analogues [60]. It bears a relative similarity with the drug eliglustat, a benzodioxin derivative bearing a pyrrolidinyl moiety used to treat Gaucher disease type 1. Eliglustat (Cerdelga™) is a ceramide mimic inhibiting the enzyme UDP-glucose ceramide glucosyltransferase (UCCG) that synthesizes glucosylceramides. It therefore reduces accumulation of these lipids in the lysosome [76]. The drug has revealed interest for the treatment of cancers, notably in combinations with immune checkpoint inhibitors [77]. It would be interesting to investigate further the bioactivity and mechanism of the action of hypercratine in this context as a potential UCCG inhibitor, in parallel with its possible action as a modulator of β2-adrenergic receptors. It is to be hoped that the phytochemical survey reported here will help and encourage phytochemists and pharmacologists to investigate further *Ruspolia* species and associated natural products.

Overall, there is a need for a more in-depth exploration of the potential for pharmacological evaluation of the compounds isolated from *Ruspolia* plants, notably from the neglected medicinal species *R. hypocrateriformis*. The plant is abundant and accessible. The main alkaloids, notably ruspolinone and hypercratine, can be obtained via efficient synthetic procedures. These pyrrolidine alkaloids deserve further studies as anti-inflammatory, anticancer, and/or antimicrobial agents, and they can serve as a template for the design of novel pyrrolidine derivatives. Pyrrolidine molecules represent an important class of medicinal products [78,79,80]. There is a need for new scaffolds and lead molecules in this family.

## 5. Conclusions

The phytochemical survey of the *Ruspolia* plant species has revealed the presence of bioactive products, mainly from the medicinal species *R. hypocrateriformis* with several pyrrolidine alkaloids and a few flavone glycosides. The main products correspond to ruspolinone and derivatives, for which there are efficient synthetic procedures to obtain the compounds and analogues. The analysis also sheds light on the alkaloid hypercratine, likely biosynthesized from ruspolinone, acting as a potential ligand to β2-adrenergic receptors. This compound and related *Ruspolia* alkaloids deserve further study to better define their mechanism of action and molecular targets. The analysis will encourage pharmacological investigations into the mode of action of *Ruspolia*-derived natural products.

## Figures and Tables

**Table 1:** *Ruspolia* species.

Accepted Names ^1^	Synonyms
*Ruspolia australis* (Milne-Redh.) Vollesen	*Ruspolia hypocrateriformis* var. australis Milne-Redh.
*Ruspolia decurrens* (Hochst. ex Nees) Milne-Redh.	*Eranthemum decurrens* Nees *Eranthemum senense* Klotzsch *Pseuderanthemum decurrens* (Nees) Radlk. *Pseuderanthemum senense* (Klotzsch) Radlk.
*Ruspolia hypocrateriformis* (Vahl) Milne-Redh.	*Ruspolia pseuderanthemoides* Lindau *Justicia hypocrateriformis* Vahl. *Eranthemum affine* Spreng. *Eranthemum hypocrateriforme* (Vahl) Sol. ex Roem. & Schult.
*Ruspolia paniculata* Benoist	
*Ruspolia seticalyx* (C.B.Clarke) Milne-Redh.	*Eranthemum seticalyx* C.B.Clarke

^1^ from World Flora Online (www.worldfloraonline.org, accessed on 3 January 2025).

**Table 2:** Calculated potential energy of interaction (ΔE) and free energy of hydration (ΔG) for the interaction of hypercratine with the β2-adrenergic receptor (β2AR) *.

Compounds	ΔE (kcal/mol)	ΔG (kcal/mol)
Hypercratine	−73.10	−73.40
Isoproterenol	−45.80	−58.60
Ritodrine	−51.45	−63.90
Salbutanol	−46.60	−52.85
Terbutaline	−46.80	−67.25

* Docking models constructed using the cryo-EM structure of β2AR (PDB: 7DHR) with the side chains of the following amino acids within the binding site were rendered fully flexible: Trp109, Asp113, Val117, Phe193, Tyr199, Phe208, Trp286, Phe290, Ile309, and Typ316. The docking procedure has been previously described [68,69].

**Table 3:** Natural products isolated from *Ruspolia hypocrateriformis* and their bioactivities.

Natural Products	Chemical Type	Plant Parts	Bioactivities	Ref.
Justicialosides A,B	Flavone glycosides	Leaves	Antioxidant activity	[18]
Chrysoeriol glycoside	Flavone glycoside	Leaves	Free radicals scavenging	[21]
Ruspolinone Norruspolinone Norruspoline N-methylruspolinone	Alkaloids	Whole plant	Anticancer and antimicrobial effects	[25,26,27]
10*H*-quindoline	Alkaloid	Leaves	Cytotoxic activity	[17]
Hypercratine	Alkaloid	Roots	Potential binding to β2-adrenergic receptors (β2AR)	[60]

## Data Availability

No new data were created.
